# Dynamic Double-Networked Hydrogels by Hybridizing PVA and Herbal Polysaccharides: Improved Mechanical Properties and Selective Antibacterial Activity

**DOI:** 10.3390/gels10120821

**Published:** 2024-12-13

**Authors:** Weidong Liu, Chuying Yao, Daohang Wang, Guangyan Du, Yutian Ji, Quan Li

**Affiliations:** 1 Tianjin Key Laboratory of Therapeutic Substance of Traditional Chinese Medicine, School of Chinese Materia Medica, Tianjin University of Traditional Chinese Medicine, Tianjin 301617, China; ericdong10033@163.com; 2 College of Materials Science and Engineering, Zhejiang University of Technology, Hangzhou 310014, China; 211122250022@zjut.edu.cn (C.Y.); 2112125158@zjut.edu.cn (D.W.); 3 Collaborative Innovation Center for Advanced Organic Chemical Materials Co-Constructed by the Province and Ministry, Ministry-of-Education Key Laboratory for the Synthesis and Application of Organic Functional Molecules, College of Chemistry and Chemical Engineering, Hubei University, Wuhan 430062, China; jyuutian@163.com

**Keywords:** polyvinyl alcohol, natural polysaccharides, double network, hydrogel, herbal medicine

## Abstract

Chinese herbal medicine has offered an enormous source for developing novel bio-soft materials. In this research, the natural polysaccharide isolated from the Chinese herbal medicine *Dendrobium* was employed as the secondary building block to fabricate a “hybrid” hydrogel with synthetic poly (vinyl alcohol) (PVA) polymers. Thanks to the presence of mannose units that contain cis-diol motifs on the chain of the *Dendrobium* polysaccharides, efficient crosslinking with the borax is allowed and reversible covalent borate ester bonds are formed. Eventually, highly dynamic and double-networked hydrogels were successfully prepared by the integration of *Dendrobium* polysaccharides and PVA. Interestingly, the introduction of polysaccharides has given rise to more robust and dynamic hydrogel networks, leading to enhanced thermal stability, mechanical strength, and tensile capacity (>1000%) as well as the rapid self-healing ability (<5 s) of the “hybrid” hydrogels compared with the PVA/borax single networked hydrogel. Moreover, the polysaccharides/PVA double network hydrogel showed selective antibacterial activity towards *S. aureus*. The reported polysaccharides/PVA double networked hydrogel would provide a scaffold to hybridize bioactive natural polysaccharides and synthetic polymers for developing robust but dynamic multiple networked hydrogels that are tailorable for biomedical applications.

## 1. Introduction

Hydrogels are three-dimensional materials capable of holding large amounts of water within their network structure [1,2,3,4,5,6]. Known for their high porosity, excellent water retention, and mechanical properties, hydrogels are considered promising artificial biomaterials and have found widespread application, particularly in biomedical fields including wound dressings, drug delivery, tissue engineering, etc. [7,8,9,10]. Currently, the hydrogel’s properties such as mechanical strength, biocompatibility, and biodegradability, to name a few, can be engineered by careful selection of building blocks, crosslinking agents, and adjustment of component ratios, which further allowed tailoring of the hydrogel functionality and performance adaptable to the application scene. Other innovative approaches, including the development of nano-composite hydrogels [11,12], double network hydrogels [13,14], and multi-interaction hydrogels [15], have also been explored to modulate the mechanical properties and functionalities of the hydrogels.

Polyvinyl alcohol (PVA) is a water-soluble polymer that is frequently used for preparing hydrogels due to its excellent solubility, low toxicity, good biocompatibility, and biodegradability [16,17,18,19,20]. Most intriguingly, the abundant hydroxyl groups on the PVA chain have allowed cross-linking with borax to form reversible borate esters and dynamic hydrogels, imparting self-healing properties for fabricating versatile soft materials. However, the mechanical strength of such single PVA-borax networked hydrogels often proved to be weak, and efforts to improve the mechanical properties are usually needed [20,21]. Recent research has demonstrated the incorporation of a polysaccharide block into the PVA-borax hydrogel system to build multiple crosslinking networks that can overcome these limitations. For instance, Zhong et al. developed a resilient and multifunctional composite PVA-borax-based hydrogel by introducing dopamine-grafted oxidized carboxymethyl cellulose and cellulose nanofibers [22]. Ma et al. constructed a robust PVA hydrogel by blending okra polysaccharides with silver nanowires in a unique sandwich structure suitable for strain-sensing devices [23]. Zhang et al. combined Aloe polysaccharides, honey, and PVA with borax, and a freeze-thaw method was used to create a hydrogel with good mechanical strength and excellent biocompatibility [24]. Apparently, more building blocks besides polysaccharides were often necessary to either reinforce the network or endow the hydrogels with desired functions. Therefore, developing simpler hybridized PVA and polysaccharides-based hydrogels with appropriate mechanical properties is highly demanded.

*Dendrobium* has long been used as a unique herb for disease treatment in Chinese herbal medicine, it is regarded as the “top one in nine immortal herbs” in China. Polysaccharides have been elucidated as a main bioactive constituent in *Dendrobium*, and they possess diversified bioactivities, including immunomodulatory [25,26], hepatoprotective [27,28], anti-inflammatory [29,30], anti-oxidant [31], anti-tumor [32], and hypoglycemic activities [33]. The *Dendrobium* polysaccharides primarily consist of glucose and mannose. Hence, the abundance of hydroxyl groups, particularly the cis-diol structure, in mannose from these polysaccharides would allow successful engagement with the chemistry of borate ester, and dynamic networked hydrogels with biofunctions can be envisioned. Previously, Gao et al. [34] have developed an environment-responsive material for postoperative adjuvant therapy by incorporating Mn^2+^-pectin microspheres into a *Dendrobium* polysaccharide hydrogel. The MnP@DOP-Gel material exhibits ROS-responsive MnP release, which induces immunogenic cell death in tumor cells and activates dendritic cells and macrophages to trigger a cascade of antitumor immune responses, thereby fulfilling a dual function. This study broadens the application of *Dendrobium* polysaccharides in drug delivery, although research into its biomedical applications, such as wound healing, selective antibacterial effects, and tissue engineering, remains limited.

**Scheme 1:**
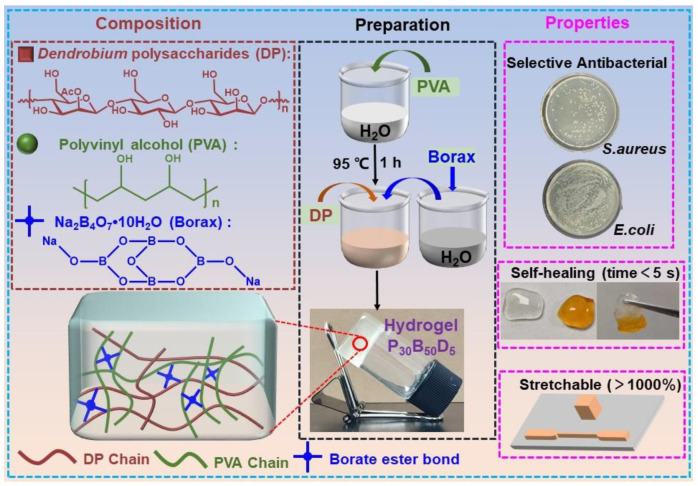
Schematic illustration of the composition, preparation, and properties of double networked PVA/DP hydrogels.

Herein, taking advantage of synthetic PVA polymers and natural *Dendrobium* polysaccharides (DP), double dynamic cross-linked PVA/DP hydrogels are prepared via the formation of the reversible borate ester bonds (Scheme 1). Such simpler dynamic hydrogels have demonstrated significantly enhanced mechanical strength and rapid self-healing capability. Moreover, the dynamic PVA/DP hydrogels showed outstanding selective antibacterial activity toward *S. aureus*. We believe the reported hybrid synthetic and natural polymeric hydrogels built by the formation of a double dynamic network will offer scaffolds for developing novel smart bio-soft materials to address the challenges in biomedical fields [35,36].

## 2. Results and Discussion

### 2.1. Preparation and Characterization of PVA/DP Hydrogels

The PVA/DP hydrogels were prepared by blending PVA (P), DP (D), and borax (B) with different mass ratios in 1 mL of deionized water at 95 °C. The feed amount for all the hydrogel building blocks is listed in Table 1. For brevity, all the hydrogels are labeled as P_x_B_y_D_z_. The subscripts X, Y, and Z represent the final concentration of each building block (mg/mL). All the hydrogels were rapidly formed and are assumed to feature a dynamic dual network where PVA and DP are crosslinked with borax through reversible borate ester bonds due to the presence of a cis-diol motif on both polymer chains. For comparison, single networked PVA/borax (P_30_B_10_, P_30_B_30_, P_30_B_50_) hydrogels were also prepared. As shown in Figure S1, the strain dependence of G′ and G″ for hydrogels P_30_B_10_, P_30_B_30_, and P_30_B_50_ was compared, and hydrogel P_30_B_50_, exhibiting superior gel strength, was selected for further experimental testing and comparison. Additionally, the G′ and G″ values of the P_30_B_50_ hydrogel are higher than those reported in the literature.

**Figure 1:**
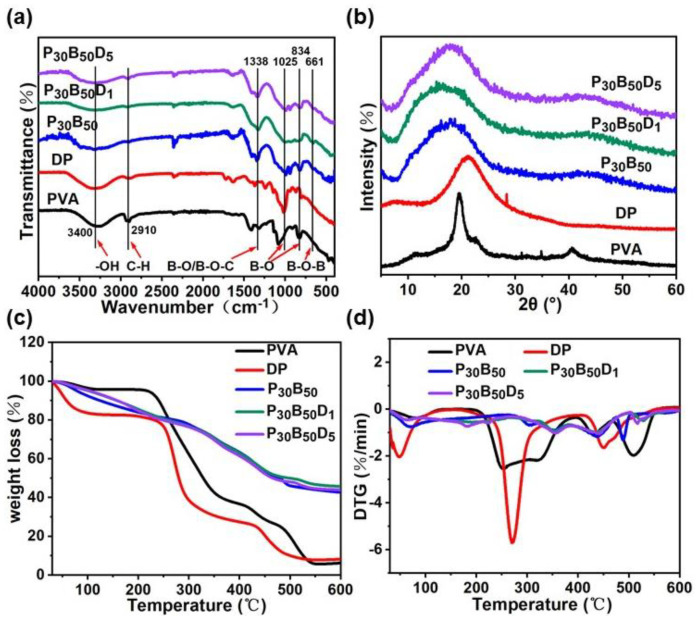
(**a**) FT−IR spectra, (**b**) XRD patterns, (**c**) TG, (**d**) DTG curves of P_30_B_50_, P_30_B_50_D_1_, and P_30_B_50_D_5_ hydrogels and their compositions. All the samples were freeze-dried before the measurement.

To validate the formation of reversible borate ester in the network, Fourier-transform infrared (FT-IR) spectroscopy was performed. As shown in Figure 1a, the hydrogels P_30_B_50_, P_30_B_50_D_1_, and P_30_B_50_D_5_ exhibit asymmetric stretching vibrations at 661, 834, and 1025 cm^−1^, which are associated with residual borate B-O stretching vibrations and B-O-B bond bending in the borate network [21,36,37]. Additionally, a narrow absorption band at 1338 cm^−1^ is attributed to the stretching vibrations of B-O and B-O-C groups [38]. All samples show a stretching vibration band at 2910 cm^−1^, corresponding to saturated C-H bonds, and a broad absorption band around 3400 cm^−1^, indicating the presence of -OH groups in polysaccharides and hydrogen bonds in the hydrogel, leading to wide absorption peaks. These results confirm the successful fabrication of a three-dimensional crosslinked network in the P_30_B_50_, P_30_B_50_D_1_, and P_30_B_50_D_5_ hydrogels through reversible borate ester bonds.

An X-ray diffraction experiment was also performed to confirm the formation of the borate ester-linked dual hydrogel network Figure 1b. The bulk PVA polymer shows its characteristic peaks at 2θ = 19.5°, 22.5°, and 40.6°, reflecting its microcrystalline structures [39,40]. Upon crosslinking with borax, these characteristic peaks disappear. A new broad diffraction peak centered at 18.5° appears for the hydrogel P_30_B_50_, suggesting that the material has a predominantly amorphous, low-crystallinity structure. Such changes in diffraction patterns indicate the disruption of the microcrystalline structure of PVA due to the newly formed dynamic borate ester-linked network [18,41,42]. Upon the addition of DP into the system, the diffraction peaks of the hydrogel P_30_B_50_D_1_ and P_30_B_50_D_5_ respectively shift to lower angles at 17.8° and 17.2°. A shoulder at 10.4° also appears for the hydrogel P_30_B_50_D_5_. Notably, the diffraction peak at 21.2° for bulk DP completely vanishes upon cross-linking with borax, suggesting that the molecular structure of hydrogel P_30_B_50_D_5_ may undergo rearrangement, thereby indicating the successful integration of DP into the double-networked hydrogels [43,44].

Having established the formation of the dynamic reversible network for the hydrogel P_30_B_50_, P_30_B_50_D_1_, and P_30_B_50_D_5_, we next examined the stability of these crosslinked hydrogels using the thermogravimetric analysis (TGA). As shown in Figure 1c, both the bulk PVA and DP polymer undergoes a rapid 85% loss of mass within 600 °C. However, only a 55% loss of mass was observed for the hydrogels P_30_B_50_, P_30_B_50_D_1_, and P_30_B_50_D_5_ within 600 °C, lower than PVA and DP polymers. As depicted in Figure 1d, the DTG curves illustrate the rate of mass loss as temperature changes [45]. The main thermal decomposition temperatures for PVA and DP are observed at 253.7 °C and 270.0 °C, respectively. Furthermore, the main decomposition temperature of hydrogel P_30_B_50_D_5_ reaches 355.4 °C, which is slightly higher than P_30_B_50_D_1_ at 352.3 °C and P_3_B_5_ at 350.1 °C. The increase in thermal stability for the P_30_B_50_D_5_ hydrogels can be attributed to the formation of a denser double hydrogel network [46]. Additionally, hydrogels P_30_B_50_D_0.1_ and P_30_B_50_D_5_ exhibit no significant weight loss at the decomposition temperature of ~270.8 °C, which is for individual PVA and DP, suggesting that hydrogels P_30_B_50_D_1_ and P_30_B_50_D_5_ exhibit a synergistic double-network effect, enhancing their thermal stability, further indicating the successful formation of a double-network hydrogel system involving both PVA and DP polymers.

**Figure 2:**
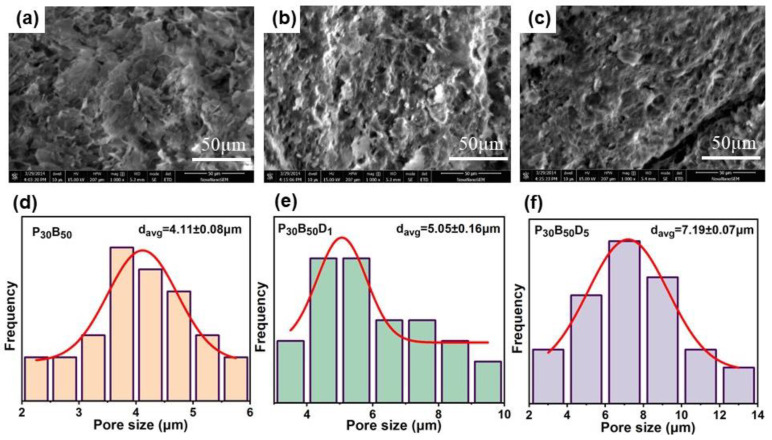
(**a**–**c**) SEM images of P_30_B_50_, P_30_B_50_D_1_, and P_30_B_50_D_5_ hydrogels. (**d**–**f**) Pore size of P_30_B_50_, P_30_B_50_D_1_, and P_30_B_50_D_5_ hydrogels.

The microstructure of all hydrogels was characterized using scanning electron microscopy (SEM), and the experimental results are shown in Figure 2. All three hydrogels in Figure 2a–c displayed characteristic three-dimensional network structures. With an increase in DP content, oriented microporous structures were formed within the hydrogels. As the mass fraction of DP increased, the micropore size of the hydrogels expanded from 4.11 ± 0.08 μm to 5.05 ± 0.16 μm, and then to 7.19 ± 0.07 μm. As a result, the borate ester bonds between PVA and borax decreased, while the borate ester bonds between DP and borax increased. Meanwhile, the hydrogen bonds between the hydroxyl groups of PVA and the DP crosslinks also increased. Consequently, the stability of the hydrogels increased, resulting in a three-dimensional network with a more uniform pored structure and morphology. The porosity of three hydrogel samples was also assessed using the liquid displacement method. As shown in Figure S2, the porosity of P_30_B_50_, P_30_B_50_D_1_, and P_30_B_50_D_5_ hydrogels exceeded 50%, with values of 81.6 ± 4.1%, 64.3 ± 1.5%, and 60.6 ± 2.4%, respectively. Porosity was negatively correlated with DP concentration, meaning higher DP concentrations resulted in lower porosity. This likely resulted from the increased DP content, which made the hydrogel network structure more compact. Newly formed borate ester bonds filled the pores of P_30_B_50_, leading to denser three-dimensional structural connections and lower porosity. The porosity results obtained by solution exchange are in contrast to the pore size trend observed by SEM, which may be attributed to the internal three-dimensional interconnected pore structure of the material [47,48].

### 2.2. Rheological Studies

**Figure 3:**
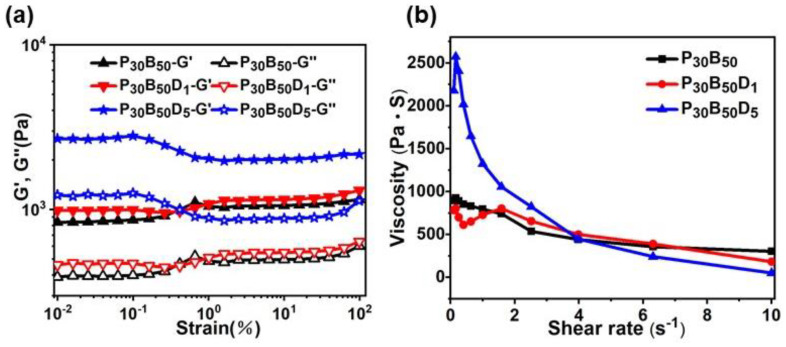
(**a**) Strain dependence of G′ and G″ of hydrogel P_30_B_50_, P_30_B_50_D_1_, and P_30_B_50_D_5_. (**b**) Shear rate−dependent viscosity of hydrogel P_30_B_50_, P_30_B_50_D_1_, and P_30_B_50_D_5_.

**Figure 4:**
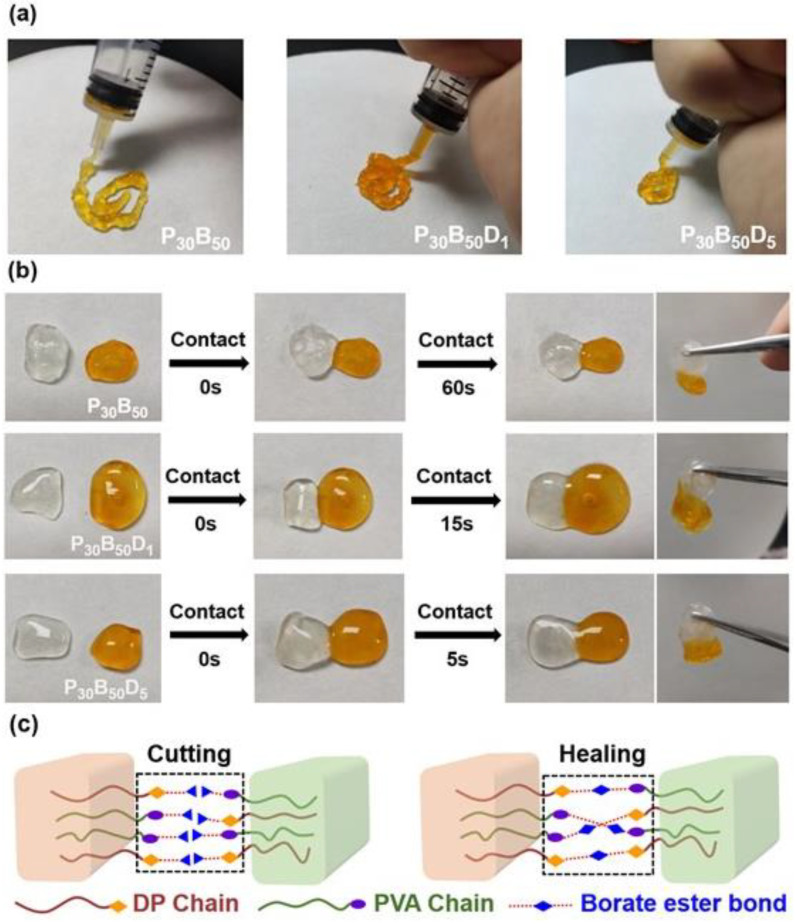
(**a**) Injectable properties of P_30_B_50_, P_30_B_50_D_1_, and P_30_B_50_D_5_ hydrogels. (**b**) Self-healing properties of P_30_B_50_, P_30_B_50_D_1,_ and P_30_B_50_D_5_ hydrogels. (**c**) Self-healing Mechanism Diagram of P_30_B_50_D_5_ hydrogels.

To probe the mechanical properties of the hydrogels, rheological studies were conducted. Initially, a dynamic strain sweep test was carried out to determine the linear viscoelastic range of the hydrogels. As depicted in Figure 3a, the storage modulus (G′) and loss modulus (G″) remained almost constant within the strain range of 0.01–100%, and the G′ is always higher than G″, suggesting that all the hydrogels consistently behave as a viscoelastic solid [49]. Remarkably, hydrogel P_30_B_50_D_5_ possessed a higher G′ than hydrogel P_30_B_50_D_1_ and P_30_B_50_, corroborating that a more robust network was formed for P_30_B_50_D_5_ [18]. To ascertain the potential injecting capabilities of such dynamic hydrogels, we measured the viscosity changes at different shear rates for these hydrogels. As shown in Figure 3b, all the hydrogels demonstrated a trend of shear-thinning behavior [50]. In particular, hydrogel P_30_B_50_D_1_ exhibited a prompter response to external shear force, likely due to the synergistic interaction between the double networks, which strengthens intermolecular interactions [51,52]. Such an observation indicates that hydrogel P_30_B_50_D_5_ should have a more dynamic network that would allow excellent injectability. Indeed, hydrogel P_30_B_50_D_5_ can be easily injected by a syringe and keeps the hydrogel state after extrusion Figure 4a [53].

### 2.3. Self-Healing Properties

Since the hydrogels P_30_B_50_, P_30_B_50_D_1_, and P_30_B_50_D_5_ have been identified as dynamic cross-linked networks, this prompted us to test their capacity for self-healing. The self-healing abilities of all the hydrogels were evaluated by a direct visual method. As shown in Figure 4b, after cutting an intact hydrogel of P_30_B_50_, P_30_B_50_D_1_, and P_30_B_50_D_5_ into two halves, they can easily self-heal into a complete one without external aid. A subsequent picking test using a tweezer shows that the healed hydrogel can support its own weight [54,55,56]. Concerning the healing time of the hydrogels, other similar PVA/Borax-based hydrogels showed a typical healing time ranging from 1 to 10 min [56,57], with some reaching 48.22 min [58], and the fastest reported healing time is 8 s [59] (Table S1). Notably, our hydrogel P_30_B_50_D_5_ demonstrated the most rapid healing time within 5 s, which can be ascribed to the formation of a denser double crosslinked network, leading to a more efficient healing process (Video S1 in Supporting Information). The self-healing mechanism of P_30_B_50_D_5_ hydrogels is depicted in Figure 4c. When the hydrogel network undergoes disruption due to external forces, borate ester bonds are cleaved. Once the split hydrogel pieces contact each other, new reversible borate ester bonds are formed at the interface, reestablishing the hydrogel network [55,60].

### 2.4. Tensile Properties

**Figure 5:**
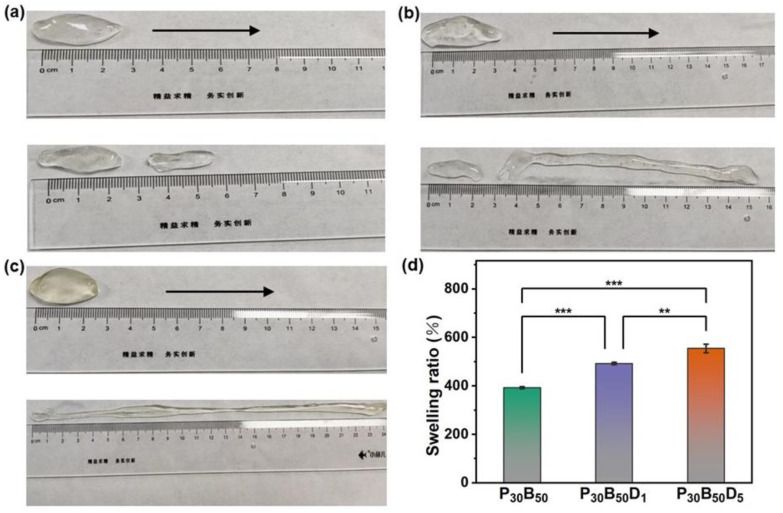
Tensile properties of (**a**) P_30_B_50_, (**b**) P_30_B_50_D_1_, and (**c**) P_30_B_50_D_5_ hydrogels. The arrow denotes the stretching direction. (**d**) Swelling ratio of P_30_B_50_, P_30_B_50_D_1_, and P_30_B_50_D_5_ hydrogels in PBS. Data are reported as means ± SD, *n* = 3 (** *p* < 0.01, *** *p* < 0.001).

The highly dynamic feature of such double-networked hydrogels prompted us to further explore their tensile performance, which was demonstrated through the direct visual observation method. The hydrogel’s tensile strength was visually evaluated by fixing both ends between the thumb and index finger and stretching it at a constant speed [61]. Figure 5a illustrates that the P_30_B_50_ hydrogel, characterized by a single cross-linked network, displays almost no tensile strength and fracture resistance when stretched in one direction. However, with the addition of a small amount of DP, the tensile strength of hydrogel P_30_B_50_D_1_ increases significantly to 600%, as shown in Figure 5b. It further reaches over 1000% with the addition of more DP for the hydrogel P_30_B_50_D_5_, as shown in Figure 5c. As shown in Table S2, most similar PVA/Borax-based hydrogels demonstrated a relatively lower tensile capability, with elongation values of 200% [56], 203.3% [62], and 275% [63]. The best-performing hydrogels reach elongation values of 792% [64] and 975% [58]. In contrast, the polysaccharide-based hydrogel developed in our study exhibits an exceptional elongation value of 1000%, indicating excellent stretchability. These initial tensile test results indicate that the introduction of DP into the P_30_B_50_ hydrogel to build a secondary network can significantly boost the overall tensile strength of the hydrogel. This tensile strength enhancement is attributed to the densified but more dynamic crosslinks within the network, which subsequently improves the hydrogel’s elasticity without compromising its structural integrity [21].

### 2.5. Swelling Ratio

Hydrogels are characterized by their inherent ability to swell, increasing in volume and mass upon water absorption. Herein, to evaluate the swelling capability of the hydrogel in the aqueous solution, phosphate-buffered saline (PBS) solution (10 mM, pH 7.4) was selected for the swelling test in light of its future potential for biomedical applications, e.g., wound dressings [21]. Figure 5d illustrates the swelling behavior of different hydrogels immersed in a PBS solution at room temperature. The swelling ratio of the P_30_B_50_D_5_ hydrogel (554.9%) is significantly higher than that of the P_30_B_50_D_1_ hydrogel (492.4%) and the P_30_B_50_ hydrogel (392.9%). This enhanced swelling behavior can be attributed to the introduction of more hydrophilic natural polysaccharide blocks into the hydrogel system, which remarkably improves hydrogel hydrophilicity. Furthermore, an increase in the DP content leads to a rise in both the quantity and size of the pores within the hydrogel network, resulting in greater pore expansion and improved water absorption capacity, and eventually boosts the swelling ratio of the P_30_B_50_D_5_ hydrogel. Considering the application of hydrogels in the biomedical field, having an appropriate swelling ability in an aqueous environment is very important. For example, many similar PVA/Borax-based hydrogels with swelling ratios from 32% [65] to 396% [66] and up to 600% [56] have demonstrated potential for wound dressing applications, as shown in Table S3. Hence, the P_30_B_50_D_5_ hydrogel with the highest swelling ratio together with the best tensile properties further guarantees its potential as a wound dressing material.

### 2.6. Water Retention Properties and In Vitro Degradation Behavior

**Figure 6:**
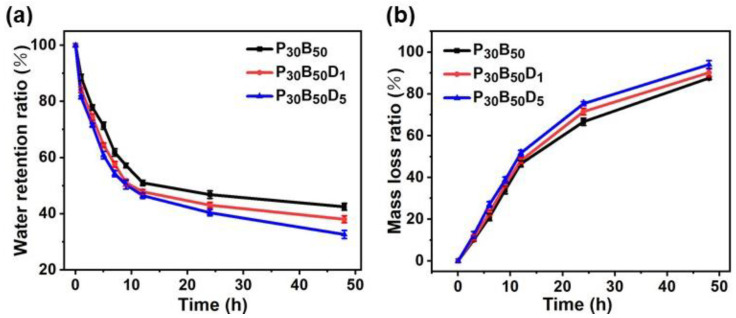
(**a**) Water retention properties of hydrogel P_30_B_50_, P_30_B_50_D_1_, and P_30_B_50_D_5_. (**b**) In vitro degradation behavior of hydrogel P_30_B_50_, P_30_B_50_D_1_, and P_30_B_50_D_5_. Data are reported as means ± SD, *n* = 3.

Figure 6a presents the water retention rates of the hydrogels. All hydrogel samples showed rapid water loss during the first 10 h. Notably, while the P_30_B_50_D_5_ hydrogel retained less water than the P_30_B_50_ hydrogel, it still maintained a sufficient moisture environment. The water retention rates exhibited an opposite trend to the swelling behavior, but shared the same underlying mechanism related to pore size. An ideal hydrogel should degrade effectively after completing its biofunctions, e.g., for wound healing [67]. Hence, the in vitro degradation behavior of the hydrogels was studied in PBS solution at pH 7.4. Figure 6b shows that the mass of all three hydrogels decreased significantly with a longer immersion time. The degradation of hydrogels would result from the physical dissolution of matrix components during hydration and the chemical cleavage of the dual-network structure [68,69].

### 2.7. Antibacterial Activity

**Figure 7:**
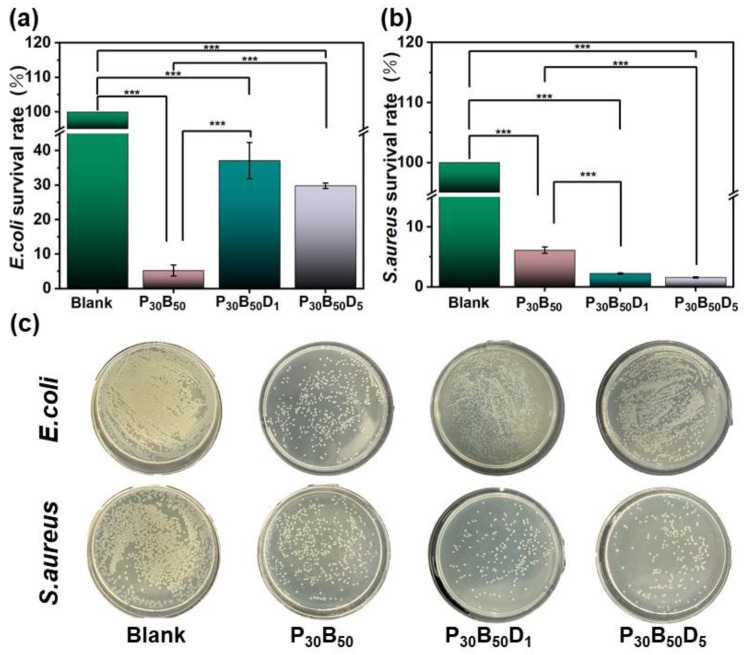
Antibacterial activity of the P_30_B_50_, P_30_B_50_D_1_, and P_30_B_50_D_5_ hydrogels. (**a**–**c**) Quantitative statistics of the bacterial killing ratio of the hydrogels against *E. coli* and *S. aureus* in the surface antibacterial activity test. Data are reported as means ± SD, *n* = 3 (*** *p* < 0.001).

To explore the application potential of such dynamic double networked and particularly natural polysaccharides-integrated hydrogels, we initially evaluate the antibacterial activity of hydrogels P_30_B_50_, P_30_B_50_D_1_, and P_30_B_50_D_5_, since hydrogels are now widely applied as wound dressings to prevent wound infections [24,70]. To this end, two representative bacterial strains, the Gram-negative *E. coli* and Gram-positive *S. aureus*, were selected. The surface antibacterial activity test was employed to evaluate the antibacterial efficacy of these hydrogels [55,71]. As shown in Figure 7, after 24 h of exposure, the survival rates of *E. coli* treated with P_30_B_50_, P_30_B_50_D_1_, and P_30_B_50_D_1_ hydrogels were 5.27%, 37.11%, and 29.81%, respectively, as shown in Figure 7a. The survival rates of *S. aureus* treated with P_30_B_50_, P_30_B_50_D_1_, and P_30_B_50_D_5_ hydrogels were only 6.12%, 2.23%, and 1.57%, respectively, as shown in Figure 7b. These results indicate that the P_30_B_50_ hydrogel exhibits stronger antibacterial properties against *E. coli* compared with the P_30_B_50_D_1_ and P_30_B_50_D_5_ hydrogels. He et al. [55] reported that treatment with a PBO1 hydrogel can reduce the survival rates of *E. coli* and *S. aureus* to 2% and 0.6%, respectively. Similarly, Yi et al. [60] reported survival rates of 15.9% for *E. coli* and 14.0% for *S. aureus* after treatment with the PB-EPL/TA@BC hydrogel. The P_30_B_50_D_5_ hydrogel prepared in this study does not demonstrate significant synergistic antibacterial effects against *E. coli* and *S. aureus*. However, the P_30_B_50_D_1_ and P_30_B_50_D_5_ hydrogels exhibit stronger antibacterial properties against *S. aureus*, suggesting a selectivity over different bacterial strains for the polysaccharide-integrated hydrogels [72,73,74]. The cell wall of *S. aureus* contains a thick peptidoglycan layer, making it susceptible to damage by specific antimicrobial agents. It has been reported that the active components in Dendrobium polysaccharide-based hydrogels may interact with the peptidoglycan layer of the cell wall of *S. aureus* to inhibit its synthesis or disrupt the cell membrane, thereby hindering bacterial growth [75,76]. In contrast, *E. coli* has a more complex cell wall structure, including an outer membrane and a lipopolysaccharide layer, which distinguishes it from *S. aureus*. Such an outer membrane may act as a barrier, preventing the entry of hydrophilic or macromolecular substances into the cell of *E. coli* [77,78]. As a result, the Dendrobium polysaccharide-based hydrogels may have difficulties penetrating the outer membrane of *E. coli*, leading to reduced antimicrobial activity. Although PVA/DP hydrogels demonstrate selective antibacterial activity, further investigation of their composition/bioactivity relationship is necessary to better understand their structural characteristics and the molecular mechanisms responsible for their antibacterial effects.

## 3. Conclusions

In summary, we have successfully prepared novel and simpler double-networked hydrogels by crosslinking *Dendrobium* polysaccharide and synthetic PVA polymers with borax. Compared with the single-networked PVA/borax hydrogel, double-networked PVA/DP hydrogels demonstrated enhanced thermostability, mechanical strength, and tensile capacity, as well as rapid self-healing ability due to the introduction of a robust but more dynamic hydrogel network. Intriguingly, such PVA/DP hydrogels showed selective antibacterial efficacy against *S. aureus* over *E. coli*. Our research highlights a novel and simpler strategy for developing double-networked hydrogels by hybridizing herbal medicine-derived polysaccharides with synthetic polymers. Considering the diversified structures and functions of the herbal-medicine-derived polysaccharides, it is possible to fabricate enormous dynamic double networked bio-soft materials with fewer building blocks while tailoring properties and performance.

## 4. Materials and Methods

### 4.1. Materials

*Dendrobium* polysaccharide (DP, ≥99.5% purity, M_w_ = 230 KDa, the molar ratio of mannose, glucose, and galactose = 37.8:21.9:1) was purchased from BoRui Saccharide Biotech Co., Ltd. (Yangzhou, China). Its structure has been characterized in previous literature (fraction DHPW1) [79]. Poly (vinyl alcohol) (PVA, 99.8 mol%, Mw~88,000, BR), borax (sodium tetraborate decahydrate, Na_2_B_4_O_7_•10H_2_O, 99.99% purity), and phosphate buffer solution (PBS, pH = 7.2–7.4) were purchased from Titan Scientific Co., Ltd. (Shanghai, China). All reagents were analytically pure and purchased from Titan Scientific Co., Ltd. (Shanghai, China). Deionized water purchased from Watsons

### 4.2. Preparation of Hydrogels

The typical procedure for the hydrogel P_30_B_50_D_5_: polyvinyl alcohol (PVA) powder (60 mg) was added to deionized water (1 mL) at 95 °C and stirred for 1 h until it was dissolved completely. *Dendrobium* polysaccharide (DP) (10 mg) was added to the PVA solution and stirred continuously at 60 °C for an additional hour to obtain a homogeneous mixture. Separately, borax powder (100 mg) was dissolved in 1 mL of deionized water at room temperature with magnetic stirring, and this borax solution was then added to the above polymeric mixture for cross-linking. In approximately 15 s, a homogeneous hydrogel was formed. A similar procedure was followed to prepare all other hydrogels except for the P_30_B_50_ hydrogel, which does not contain DP.

### 4.3. Fourier-Transform Infrared Spectroscopy (FT-IR)

The FT-IR spectra of the hydrogel samples were recorded on a Fourier-transform infrared (FT-IR) spectrometer (Bruker Tensor II, Erlangen, Germany). The recorded wavelength range is between 400–4000 cm^−1^. Each sample was scanned 32 times.

### 4.4. Powder X-Ray Diffractometer (PXRD)

The crystalline structures of the hydrogels were analyzed on an XRD diffractometer (Rigaku D/max-IIIA, Osaka, Japan) with Cu Kα radiation (λ = 0.154 nm), and the spectra were recorded from 5–60° (*2θ*) with a scanning speed of 5°/min.

### 4.5. Thermogravimetric Analysis (TGA)

The thermal stability of the hydrogels was analyzed using a Netzsch TG 209 F3 instrument (NETZSCH company, Selb, Germany). A sample with a mass of 5~10 mg was placed in an alumina crucible and heated to 600 °C with a heating rate of 5 °C /min and a nitrogen flow rate of 20 mL/min.

### 4.6. Scanning Electron Microscopy (SEM)

The microstructure of the hydrogel was investigated using a scanning electron microscope (SEM, JSM7100F, JEOL, Tokyo, Japan). Prior to observation, all samples were freeze-dried and sputtered with gold. For the analysis of pore size distribution, Nano Measurer 1.2 software was used. A minimum of 30 pores was selected for each measurement to ensure statistical reliability.

### 4.7. Porosity of Hydrogel

The porosity of the PVA/DP hydrogels was determined using the solvent displacement method. Initially, the hydrogels were freeze-dried and weighed (M_d_). The freeze-dried samples were then immersed in anhydrous ethanol until complete saturation and weighed again (M_f_). The volume of ethanol consumed (V_s_) was recorded during this process, and the procedure was repeated three times for each test. Additionally, the volume change of ethanol and the ethanol density (ρ) were recorded, and the porosity of the hydrogels was calculated using the appropriate equation [47,48]:

Porosity ratio (%) = [(M_f_ − M_d_)/(ρ × V_s_)] × 100

Data are reported as means ± SD, *n* = 3 (** *p* < 0.01, *** *p* < 0.001).

### 4.8. Rheological Property

The rheological behavior of the hydrogel was investigated by a rheometer (TA DHR-2, Waters-TA Instruments, New Castle, DE, USA). Rheological tests were conducted over a strain range of 0.01–200% at an angular frequency of 10 rad/s to assess changes in both the storage modulus and the loss modulus.

### 4.9. Macroscopic Self-Healing Test of Hydrogels

Two hydrogel samples, each with a diameter of 10 mm, were prepared in advance. To visually demonstrate the self-healing capability, one of the hydrogels was dyed with methyl orange, and then sliced into two pieces. The two pieces of the hydrogel were brought to contact and placed at 25 °C for self-healing [60]. To evaluate the strength of the repaired hydrogel, a tweezer was used to lift one corner of the sample to test if it could sustain its own weight. Photographs were taken at different stages of the healing process to document its progression.

### 4.10. Tensile Property

The hydrogel’s tensile strength was visually evaluated by fixing both ends between the thumb and index finger and stretching it at a constant speed [80,81].

### 4.11. Swelling Behavior

The swelling behavior of the hydrogels was studied by the gravimetric method [21]. The hydrogel was dried to a constant weight to obtain its dry weight (M_s_). Subsequently, the dried hydrogel samples were immersed in a phosphate-buffered saline (PBS) solution (10 mM, pH 7.4) until equilibrium was reached. Finally, excess surface water was absorbed using filter paper, and the swollen hydrogel was weighed (M_e_). The swelling ratio of the hydrogel was calculated using the following formula:

Swelling ratio (%) = [(M_e_ − M_s_)/M_s_] × 100

Data are reported as means ± SD, *n* = 3 (** *p* < 0.01, *** *p* < 0.001).

### 4.12. Water Retention Properties

First, the three hydrogel samples were dried in a freeze-dryer until all moisture was removed(The model of the freeze dryer is ZLGJ-10, Tianjin Kenuo Instrument Equipment Co., Ltd., Tianjin, China). The dried hydrogel samples were then immersed in 10 mM PBS solution at pH 7.4 until swelling equilibrium was reached. Excess surface moisture was removed using filter paper, and the initial weight (M_0_) was recorded [21]. The samples were placed at room temperature for 48 h and weighed at different time points (M_t_). The water retention ratio of the hydrogels was calculated using the following formula:

Water retention ratio (%) = (M_t_/M_0_) × 100

Data are reported as means ± SD, *n* = 3 (** *p* < 0.01, *** *p* < 0.001).

### 4.13. In Vitro Degradation Behavior

The in vitro degradation behavior of the three hydrogels in PBS solution at pH 7.4 was investigated using the gravimetric method [21]. A certain mass of hydrogel (denoted as M_1_) was immersed and incubated in PBS solution at pH 7.4 at 37 °C. At regular intervals, the hydrogel was removed, and surface moisture was blotted with filter paper before weighing. The mass of the hydrogel was recorded as M_2_ after each removal. The mass loss ratio of the hydrogel was calculated using the following formula:

Mass loss ratio (%) = [(M_1_ − M_2_)/M_1_] × 100

Data are reported as means ± SD, *n* = 3 (** *p* < 0.01, *** *p* < 0.001).

### 4.14. Antibacterial Test

100 µL of either *E. coli* or *S. aureus* stock culture were added to 10 mL of Mueller-Hinton (MH) liquid medium. The mixture was incubated at 37 °C while shaking for 24 h, after which the bacterial suspension was stored at 4 °C as the stock solution for *E. coli* or *S. aureus*. This stock solution was diluted to 10^7^ CFU/mL and 10^6^ CFU/mL using sterile PBS to prepare working bacterial solutions. The samples were heated in a water bath at 80 °C for 1 h, and then they were allowed to cool for 3 h. Each sample was subsequently cut into eight pieces, each weighing 100 mg, and placed in a laminar flow hood. The samples were exposed to UV light for 60 min. Using the surface drop method, 100 mg of each sample was placed into wells of a 24-well plate. Onto the surface of each sample, 10 µL of the 10^7^ CFU/mL bacterial working solution was added. The plate was sealed and incubated at 37 °C for 2–3 h. After incubation, the samples were washed with 1 mL of Mueller-Hinton (MH) liquid medium. Next, 100 µL of the wash was transferred to agar plates, with three replicates prepared for each sample. The plates were incubated for 24 h, and the colonies were photographed for documentation and counted to determine the antibacterial rate. The final results were expressed as the percentage of bacterial inhibition [55]. Data are reported as means ± SD, *n* = 3 (** *p* < 0.01, *** *p* < 0.001)

## Figures and Tables

**Table 1:** Feed amount of hydrogels.

Samples	PVA (mg)	Borax (mg)	DP (mg)	Water (mL)
P_30_B_50_	60	100	0	2
P_30_B_50_D_1_	60	100	2	2
P_30_B_50_D_5_	60	100	10	2

## Data Availability

The original contributions presented in the study are included in the article and Supplementary Material, further inquiries can be directed to the corresponding authors.

## Supplementary Materials

The following supporting information can be downloaded at: https://www.mdpi.com/article/10.3390/gels10120821/s1, Figure S1: Strain dependence of G′ and G″ of hydrogel P_30_B_10_, P_30_B_30_, and P_30_B_50_; Figure S2. Porosity of hydrogel P_30_B_50_, P_30_B_50_D_1_, and P_30_B_50_D_5_; Table S1: Comparison of self-healing time of similar PVA-based hydrogels [56,57,58,59]; Table S2: Comparison of tensile properties of similar PVA-based hydrogels [56,58,62,63,64]; Table S3: Comparison of the swelling ratio of similar PVA-based hydrogels [56,65,66]. Video S1: Illustration of self-healing property of the hydrogel P30B50D5.
