# Supplementary material for: Dynamic Double-Networked Hydrogels by Hybridizing PVA and Herbal Polysaccharides: Improved Mechanical Properties and Selective Antibacterial Activity
Source: Gels. 2024 Dec 13;10(12):821. doi: 10.3390/gels10120821 (PMC11675697; doi:10.3390/gels10120821)
Supplement: Supplementary file 1 [file gels-10-00821-s001.zip › gels-3323697-Supplementary Materials .pdf]

Supporting information

Dynamic Double-Networked Hydrogels by Hybridizing PVA and Herbal Polysaccharides: Improved Mechanical Properties and Selective Antibacterial Activity

Weidong Liu[1], Chuying·Yao[2], Daohang Wang[2], Guangyan Du[2*], Yutian Ji[3], Quan·Li[1*]

[1] Tianjin Key Laboratory of Therapeutic Substance of Traditional Chinese Medicine, School of Chinese Materia Medica, Tianjin University of Traditional Chinese Medicine, Tianjin 301617, P.R. China.

[2] College of Materials Science and Engineering, Zhejiang University of Technology, Hangzhou 310014, P.R. China.

[3] Collaborative Innovation Center for Advanced Organic Chemical Materials Co-constructed by the Province and Ministry, Ministry-of-Education Key Laboratory for the Synthesis and Application of Organic Functional Molecules, College of Chemistry and Chemical Engineering, Hubei University, Wuhan 430062, P.R. China.

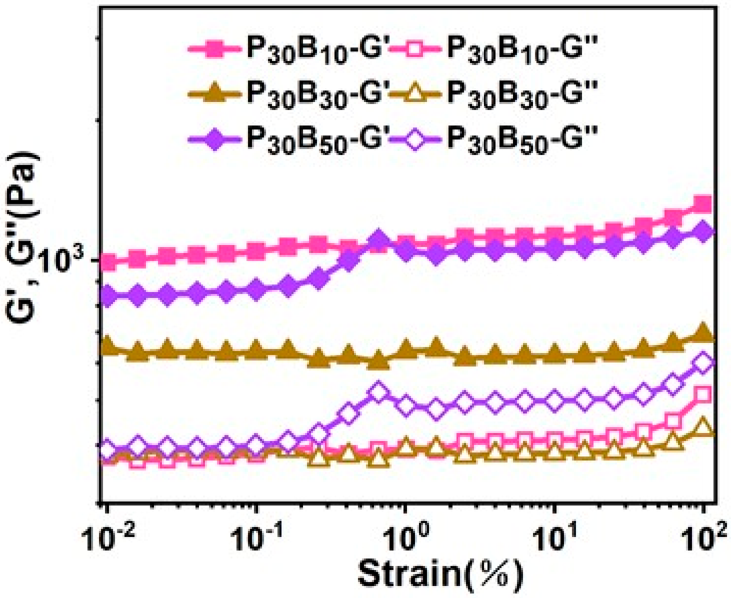

Fig. S1. Strain dependence of G′ and G′′ of hydrogel $P_{30}B_{10}$, $P_{30}B_{30}$, and $P_{30}B_{50}$.

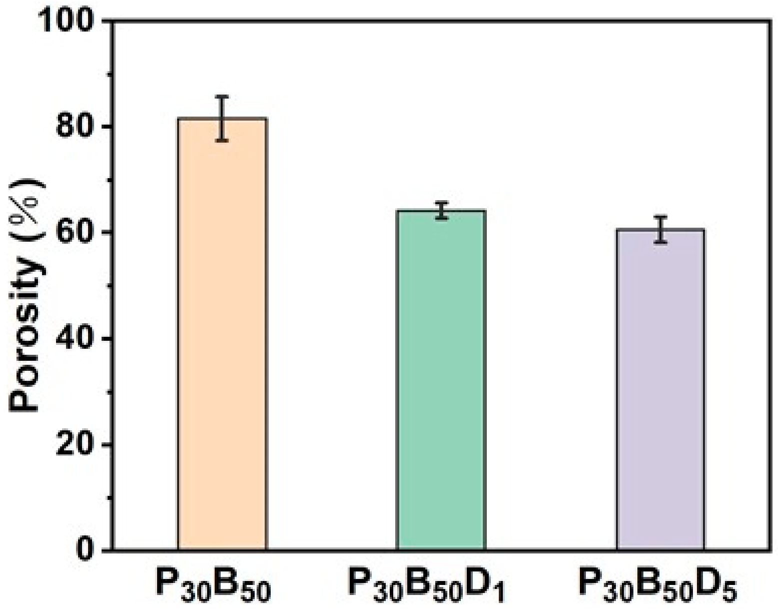

Fig. S2. Porosity of hydrogel $P_{30}B_{50}$, $P_{30}B_{50}D_1$, and $P_{30}B_{50}D_5$.

Table S1. Comparison of material self-healing time

Samples	Material composition	Self-healing time	Ref.
C-OSSPG$_{0.2}$/BP	PVA, Borax, C-OSSPG	1min	[56]
PVA/BNNS-NH_2	PVA, Borax, NNS-NH_2	10min	[57]
PVA-B-CmChi	PVA, Borax, CMC	48.22min	[58]
CTPB	PVA, Borax, CMC, TA	8s	[59]
$P_{30}B_{50}D_5$	PVA, Borax, DP	**5s**	This work

Table S2. Comparison of tensile properties of materials

Samples	Material composition	stretch rate(%)	Ref.
C-OSSPG$_{0.2}$/BP	PVA, Borax, C-OSSPG	200	[56]
PVA/G/B	PVA, Borax, gelatin	203.3	[62]
PBS	PVA, Borax, sucrose	275	[63]
PVA/CMC/PAM/Borax	PVA, CMC, PAM, Borax	792	[64]
PVA-B-CmChi	PVA, Borax, CMC	975	[58]
$P_{30}B_{50}D_5$	PVA, Borax, DP	**1000**	This work

Table S3. Comparison of material swelling ratio

Samples	Material composition	Swelling ratio(%)	Ref.
Honey/PVA hybrid hydrogel	PVA, Borax, Honey	32	[65]
GPB	PVA, Borax, Gelatin, PEDGE	396	[66]
C-OSSPG$_{0.2}$/BP	PVA, Borax, C-OSSPG	600	[56]
$P_{30}B_{50}D_{5}$	PVA, Borax, DP	**554.9**	This work
